# Efficacy of herbal medicine TJ-14 for acute radiation-induced enteritis: a multi-institutional prospective Phase II trial

**DOI:** 10.1093/jrr/rrz025

**Published:** 2019-11-06

**Authors:** Taro Murai, Masayuki Matsuo, Hidekazu Tanaka, Yoshihiko Manabe, Taiki Takaoka, Kae Hachiya, Takahiro Yamaguchi, Shinya Otsuka, Yuta Shibamoto

**Affiliations:** 1 Department of Radiology, Nagoya City University Graduate School of Medical Sciences, Nagoya, Japan; 2 Department of Radiation Oncology, Gifu University Graduate School of Medical Sciences, Gifu, Japan; 3 Department of Radiology, Japan Community Healthcare Organization (JCHO) Chukyo Hospital, Nagoya, Japan; 4 Department of Radiology, Okazaki City Hospital, Okazaki, Japan

**Keywords:** acute radiation-induced enteritis, TJ-14 (hangeshashinto, Ban Xia Xie Xin Tang), pelvic radiotherapy, cervical cancer, herbal medicine

## Abstract

The purpose of this multi-institutional Phase II trial study was to prospectively investigate the efficacy of the herbal medicine TJ-14 for acute radiation-induced enteritis (ARE). TJ-14 was administered orally as a first-line treatment for ARE. The primary end point was efficacy at 1 week. The secondary end points were: (i) the efficacy of TJ-14 at 2 and 3 weeks after its administration, (ii) the quality of life score (FACT-G) at 1, 2 and 3 weeks after its administration, and (iii) adverse events. If the efficacy of TJ-14 was observed in eight patients or fewer, its efficacy was rejected. Results: Forty patients receiving pelvic radiotherapy were enrolled. Of these, 22 developed ARE and received TJ-14. Among these, 19 had cervical cancer and 9 received chemoradiotherapy. TJ-14 efficacy was shown in 19 out of the 22 patients (86%). Stool frequency per day at 1 week significantly decreased (mean ± SD: 4.9 ± 2.1 vs 3.7 ± 1.9, *P* = 0.02). This effect continued at 2 (2.2 ± 1.4, *P* = 0.004) and 3 weeks (2.1 ± 0.9, *P* = 0.05). Thirteen out of the 22 patients (59%) continued TJ-14 until the end of radiotherapy. FACT-G score deterioration was not observed after the administration of TJ-14. Grade 1 hypokalemia was observed in 4 patients, and Grade 1 constipation in 3. We concluded that TJ-14 is sufficiently promising to be examined in a Phase III trial. A randomized controlled trial is currently being planned.

## INTRODUCTION

Pelvic radiotherapy is generally employed as a standard treatment for gynecological, genitourinary, gastrointestinal and other cancers [1, 2]. Despite advances in radiation oncology technologies, including intensity-modulated radiotherapy and 3D radiotherapy, acute radiation enteritis (ARE) remains a major adverse effect in patients receiving pelvic irradiation [3, 4]. This toxicity is observed in 20–70% of patients [1, 2]. Irradiation induces crypt epithelial cell death, which, in turn, triggers an acute inflammatory response and ARE symptoms: diarrhea, abdominal pain, bleeding, and malnutrition. Toxicity impairs digestive functions, and seriously affects nutrition and the quality of life of patients. Consequently, ARE reduces patient treatment compliance, increases the cost of care, and may alter therapeutic results by increasing overall treatment times [5]. Treatments for ARE are mostly supportive and non-specific.

TJ-14 (Hangeshashinto in Japanese, Ban Xia Xie Xin Tang in Chinese), a traditional herbal medicine used in East Asia [6, 7], is composed of seven crude herbs in fixed proportions (Supplemental Material 1). This agent has been used empirically in the treatment of various gastrointestinal disorders (including dyspepsia, nervous gastritis, gastrasthenia, and stomatitis). In Japan, many cancer patients have been treated with traditional herbal medicine to manage the adverse events of treatment, because these agents are considered to be less expensive and to have fewer adverse effects than other drugs. We also have experienced the notable therapeutic effect of TJ-14 on ARE in some patients. In rodent models, TJ-14 induces a number of anti-inflammatory responses [7, 8]. These pharmacological effects may inhibit ARE symptoms. Based on these clinical experiences and the biological activities of TJ-14, we hypothesized that TJ-14 could be a first-line treatment for ARE. However, the clinical evidence is not yet sufficient for it to be used in other countries. Therefore, we performed this prospective clinical trial to examine whether TJ-14 could be a treatment option for ARE. This study attempted to evaluate whether a randomized trial of TJ-14 would be feasible.

## METHODS

### Study design and treatment protocol

**Fig. 1.**
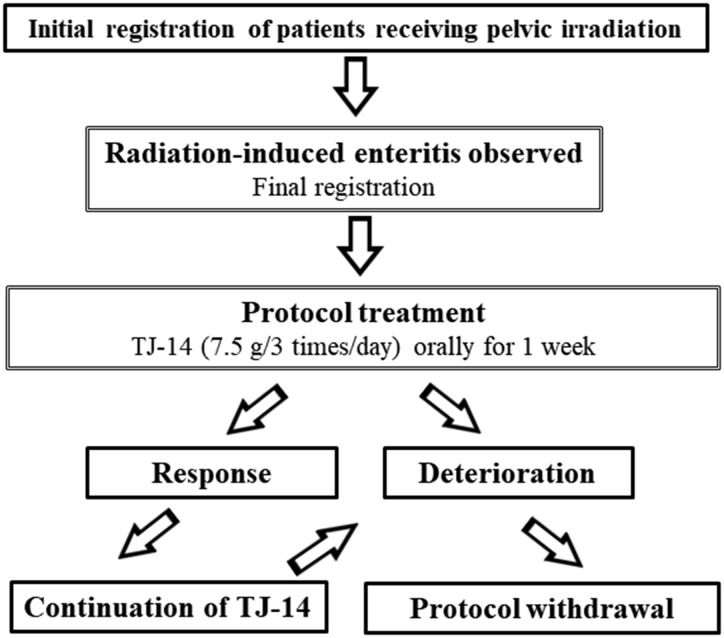
Study design.

A prospective, multi-institutional, Phase II trial was performed in four facilities (Supplemental Material 2). The study design is shown in a flow chart (Figure 1). Patients meeting the initial criteria detailed in the next paragraph were registered before the initiation of radiotherapy. The medical history, baseline diarrhea status, physical examination, blood count, biochemical tests and serum electrolytes, and quality of life score [functional assessment of the cancer therapy–general (FACT-G) score] of patients were obtained before the initiation of radiotherapy. When ARE occurred, TJ-14 was administered as a first-line treatment. These patients were finally analyzed. When the treatment protocol was effective, TJ-14 was continued thereafter until symptoms worsened or 2 months after the initiation of radiotherapy. The hypothesis was that TJ-14 is effective for ARE. If its efficacy was not demonstrated in the threshold number or fewer patients, the hypothesis was rejected.

### Initial registration criteria, ARE definition, and protocol treatment

Initial registration criteria included: (i) receiving whole-pelvis irradiation (total dose ≥40 Gy, dose per fraction ≥1.5 Gy, irradiation field covering the presacral and internal inguinal lymph node areas), (ii) Karnofsky performance status index ≥ 70, (iii) age ≥ 20 years, (iv) serum potassium level ≥ 3.5 mEq/dl, and (v) possibility of the oral administration of TJ-14. Exclusion criteria were: (i) aldosteronism, (ii) the usage of another herbal medicine, another anti-diarrhea agent, enteral nutrition agent, opioid, or steroid, (iii) an allergy to herbs, (iv) symptomatic brain metastases, (v) systemic inflammatory condition, (vi) colostomy, (vii) dementia or other serious psychological disease, (viii) pregnancy, or (ix) a previous history of pelvic and abdominal radiotherapy.

The diarrhea grading system based on common terminology criteria for adverse events (CTCAE) v. 4.0 (Table 1) was used to assess the severity of ARE. When Grade 1 or higher ARE continued for 2 days or longer, patients were finally registered and treated with TJ-14. TJ-14 was administered orally at a dose of 2.5 g three times per day (for a total daily dose of 7.5 g). No other prophylactic treatment for diarrhea was allowed.

**Table 1.** Diarrhea grading system

Grade	Symptom
1	Increase of 2–3 stools per day over the baseline
2	Increase of 4–6 stools per day over the baseline
3	Increase of ≥7 stools per day over the baseline
4	Life-threatening complications; urgent intervention indicated
5	Death

### Follow-up and end points

The primary end point was the efficacy proportion of TJ-14 1 week after administration. Aggravation in the ARE grade during TJ-14 administration was defined as treatment failure. Conversely, the absence of failure was considered efficacy of TJ-14. For example, when the stool number increased from 3 per day (baseline plus 2, Grade 1) to 5 (baseline plus 4, Grade 2) after TJ-14 administration, the protocol treatment was judged to have failed. On the other hand, even when the stool number increased from 3 (baseline plus 2, Grade 1) to 4 (baseline plus 3, Grade 1), TJ-14 was judged to have been effective. Secondary end points were: (i) the efficacy of TJ-14 2 and 3 weeks after the initiation of the treatment protocol, (ii) the FACT-G score 1, 2 and 3 weeks after the administration of TJ-14, and (iii) adverse events evaluated with CTCAE v. 4.0. These ARE symptoms were assessed using a self-reporting system by patients every day starting from registration (Supplemental Material 3). The efficacy of TJ-14 was reviewed every week. Any adverse event, whether related or unrelated to the study drug, was reported with the date, severity and outcome. All adverse events were followed until they resolved.

### Statistical analysis

The sample size was calculated by applying A’Hern’s formula. Assuming that the proportion of efficacy was 45% compared with 20% for the placebo, at least 21 patients were required for a Type 1 error of 5% and Type 2 error of 20%. If the efficacy of TJ-14 was only demonstrated in 8 or fewer out of 21 patients enrolled in this study, its efficacy was rejected. Comparisons between each continuous variable were made using the paired *t*-test. All statistical tests were two-sided. All analyses were performed in R version 3.0.0 for Windows.

### Ethics

Study data were obtained in accordance with the Declaration of Helsinki, and the study protocol was approved by the ethics review board of each institution. This trial was registered at UMIN Clinical Trials Registry in Japan (UMIN000022110). This study was designed by collaboration between Nagoya Çity University and Gifu University. All patients were given a written explanation of the study protocol and provided their written informed consent before radiotherapy.

## RESULTS

### Patient characteristics and treatment

Between September 2015 and April 2017, 40 patients were initially registered in this trial. Among them, ARE was observed in 22 patients. The last 2 patients were registered simultaneously. Patient and treatment details are summarized in Tables 2 and 3 and in Supplemental Material 2. The Karnofsky performance status was 100 in all patients. Intensity-modulated radiotherapy was delivered using a rotational or multiple static field technique. Conventional 4-field box irradiation was used in 3D radiotherapy. Patients with cervical cancer or prostate cancer received the standard whole-pelvis radiotherapy, in which the target volume covered the primary tumor, visible nodal lesion, and prophylactic lymph node area, including the common iliac, external iliac, internal iliac, obturator, and presacral node areas [9, 10]. One patient (Table 3, No. 21) received para-aortic irradiation. One patient (Table 3, No. 20) received boost irradiation to the primary tumor with 12 Gy in 6 fractions instead of brachytherapy. Cisplatin was administered weekly to 8 of these cervical cancer patients during the radiotherapy. The target volume in the rectal cancer treatment included the primary tumor and nodal regions, including the common iliac, internal iliac, obturator, and presacral nodes.

**Table 2.** Patient characteristics

		Patient No. (Total No. = 22)
Age	(Mean + SD^a^) (range)	64.2 ± 14.6 (35–87)
Male:Female		1:21
Cancer type (cervical:rectal:prostate)		19:2:1
Chemotherapy (+:−)		9:13
3DCRT^b^:IMRT^c^		9:13
Total dose^d^ (Gy) (<50:50–60:>60)		5:13:4
Brachytherapy (+:−)		11:9

^a^Standard deviation

^b^3D radiotherapy

^c^intensity-modulated radiotherapy,

^d^total external irradiation dose.

**Table 3.** Treatment characteristics

No.	Age	Sex	Cancer	S	Regimen	Modality	WP		Boost		BT
							Gy/	fr	Gy/	fr	
1	54	F	R		FP	3DCRT	50.4/	28			+
2	76	M	P			3DCRT	50.4/	28	18/	9	
3	82	F	C	+		3DCRT	45/	25			
4	73	F	C		CDDP	3DCRT	50.4/	28			+
5	79	F	C			3DCRT	50.4/	28			+
6	35	F	C			IMRT	50.4/	28			+
7	62	F	C		CDDP	IMRT	50.4/	28			+
8	46	F	C		CDDP	IMRT	50.4/	28			+
9	68	F	C	+		IMRT	50.4/	28			
10	51	F	R	+		IMRT	44/	22	22/	11	
11	74	F	C			IMRT	50.4/	28			+
12	66	F	R	+		IMRT	44/	22	20/	10	
13	58	F	C	+	CDDP	IMRT	50.4/	28			
14	84	F	C			IMRT	50.4/	28			+
15	87	F	C			3DCRT	50.4/	28			+
16	64	F	C		CDDP	3DCRT	50/	25			+
17	78	F	C			3DCRT	50/	25			+
18	61	F	C	+		IMRT	45/	25			
19	48	F	C	+		IMRT	45/	25			
20	73	F	C		CDDP	3DCRT	44/	22	12/	6	
21	48	F	C	+	CDDP	IMRT	45/	25			
22	45	F	C	+	CDDP	IMRT	45/	25			

F = female, M = male, R = rectal cancer, P = prostate cancer, C = cervical caner, S = postoperative irradiation, BT = brachytehrapy, FP = concurrent cisplatin + 5-FU, WP = whole-pelvis irradiation, CDDP = concurrent cisplatin, 3DCRT = 3D radiotherapy with 4 fields, IMRT = intensity-modulated radiotherapy, Boost = boost irradiation to the primary tumor.

### Efficacy of TJ-14

Twenty-two patients developed ARE. The ARE grade was 1 in 14, 2 in 7, and 3 in 1. Among them, ARE aggravation (Grade 1 to Grade 2) was seen in 3 patients. Disappearance of the symptom (Grade 1or 2 to Grade 0) was observed in 17, stool number decrease (Grade 2 to Grade 1) was observed in 1, and absence of ARE worsening (Grade 2 to Grade 2) was seen in 1. Thus, the efficacy of TJ-14 was observed in 19 out of the 22 patients (86%). Of these 19 patients, 2 required other anti-diarrheal agent due to continuous watery stool. Thus, 17 patients (77%) were satisfied with TJ-14 at 1 week after TJ-14 administration.

**Fig. 2.**
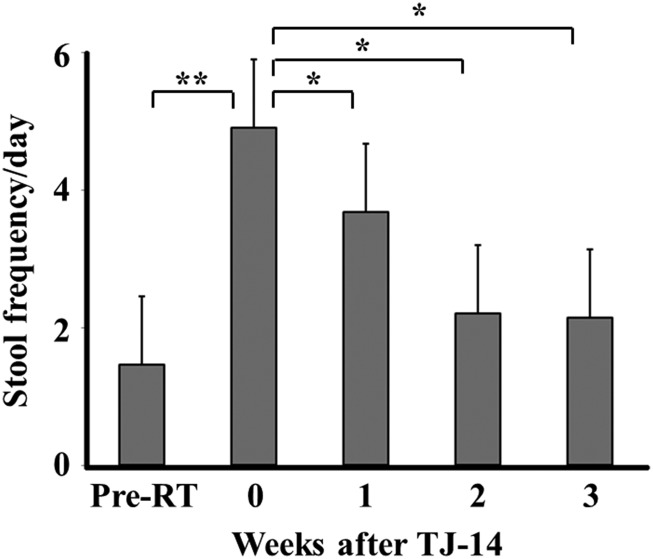
Stool frequencies before and after the administration of TJ-14. Pre-RT = Before radiation therapy, *P < 0.05, **P < 0.0005.

Stool frequencies before and after the administration of TJ-14 are shown in Figure 2. Stool frequency per day at 1 week significantly decreased [mean ± standard deviation (SD): 4.9 ± 2.1 vs 3.7 ± 1.9, *P* = 0.02]. This effect continued at 2 weeks (vs 2.2 ± 1.4, *P* = 0.004) and 3 weeks (vs 2.1 ± 0.9, *P* = 0.05) (Figure 2). Thirteen out of 22 patients (59%) continued TJ-14 until the completion of pelvic radiotherapy. The agent was discontinued in 2 patients for Grade 1 hypokalemia. Another 2 patients withdrew because of deterioration of ARE.

**Fig. 3.**
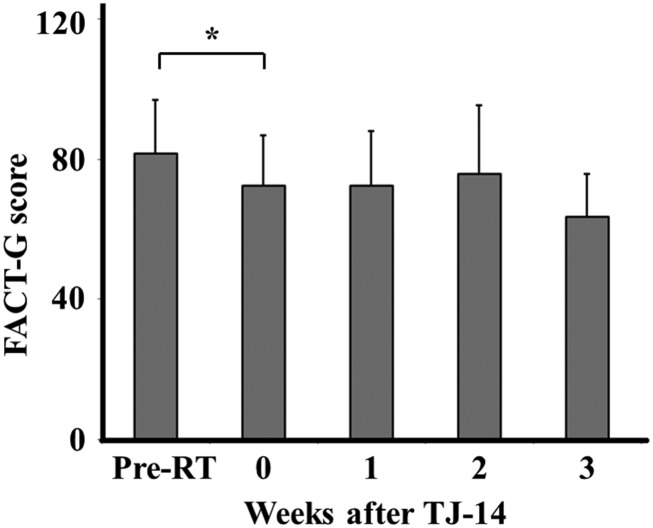
FACT-G scores before and after the administration of TJ-14. Pre-RT = Before radiation therapy, *P < 0.05.

**Fig. 4.**
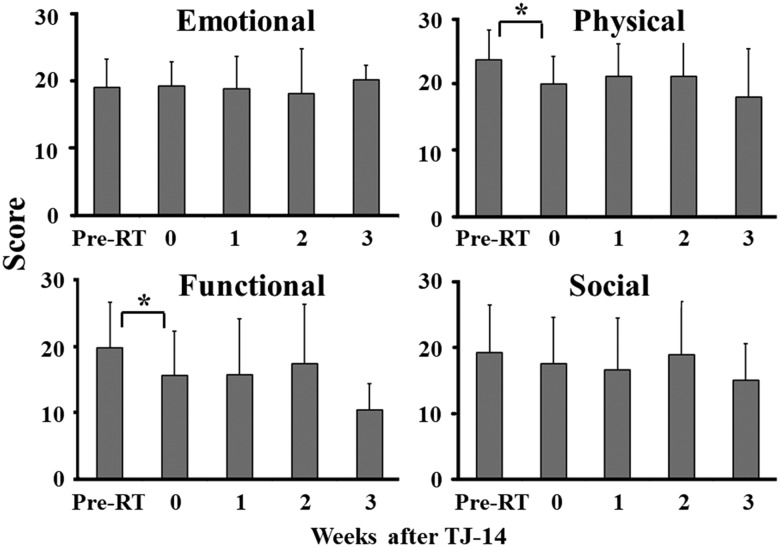
Details of FACT-G scores before and after the administration of TJ-14. Pre-RT = Before radiation therapy, *P < 0.05.

Before the radiation treatment, the mean FACT-G score was 81.8 ± 15.1 (mean ± SD). At the time of ARE development, the score deteriorated (72.5 ± 14.3, *P* = 0.009), but plateaued after the administration of TJ-14 (Figure 3). Functional and physical scores mainly changed after ARE (Figure 4).

### Adverse events

Details of adverse events are summarized in Table 3. Three patients developed Grade 2 or 3 leukopenia and 1 developed Grade 2 anemia. However, these patients received cisplatin-based chemotherapy for cervical cancer. Thus, the relationship between these adverse events and TJ-14 was unclear. Grade 1 hypokalemia was observed in 4 patients, and Grade 1 constipation in 3. Grade 2 or higher non-hematological events were not observed (Table 4).

**Table 4.** Adverse events

	Toxicity (grade) (Total No. = 22)				
	1	2	3	4	5
Abdominal pain	1	0	0	0	0
Abdominal distention	3	0	0	0	0
Constipation	3	0	0	0	0
Fecal incontinence	0	0	0	0	0
Nausea	1	0	0	0	0
Vomiting	0	0	0	0	0
Fatigue	8	0	0	0	0
Fever	0	0	0	0	0
Weight loss	0	0	0	0	0
Allergic reaction	0	0	0	0	0
Muscle weakness	0	0	0	0	0
White blood cells decreased	0	2	1	0	0
Platelet count decreased	0	0	0	0	0
Anemia	0	1	0	0	0
Creatinine increased	0	0	0	0	0
Hypokalemia	4	0	0	0	0
ALP^a^ increased	0	0	0	0	0
AST^b^ increased	0	0	0	0	0
Blood bilirubin increased	0	0	0	0	0

^a^Alkaline phosphatase.

^b^Aspartate aminotransferase.

## DISCUSSION

Although many clinical trials have attempted to evaluate various strategies to prevent ARE symptoms, a standard treatment has not yet been established. Cao *et al.* [11] conducted a meta-analysis of 13 randomized controlled trials involving 979 participants to examine whether glutamine prevents ARE symptoms. The findings obtained indicated that glutamine intake failed to improve the severity of ARE. Demers *et al.* [12] conducted a randomized double-blind control trial of 229 patients receiving probiotics as prophylaxis during pelvic radiotherapy. Three groups (placebo, standard-dose probiotics, and high-dose probiotics) were compared. The primary end point was the ability to prevent ARE. However, the study did not prove the hypothesis. Although another trial showed a significant improvement in diarrheal symptoms and a decrease in the use of anti-diarrheal medication, the study design and number of patients were not sufficient to fully advocate probiotics at this time. Therefore, the radioprotective effects of probiotics remain controversial. In addition, previous studies evaluated the different formation of aminosalicylates, including balsalazine, sulfasalazine, olsalazine and mesalazine. Kilic *et al.* [13] reported that the incidence rates of ARE in groups receiving sulfasalazine and placebo were 55% and 86%, respectively; sulfasalazine reduced the incidence of ARE in pelvic radiotherapy. On the other hand, a recent prospective study was halted after the planned interim analysis of 73 participants [14]. ARE occurred in 29% of patients receiving sulfasalazine and in 11% of the placebo group. These findings contradict one another. Thus, it currently remains unclear whether sulfasalazine is applicable as prophylaxis against ARE. Furthermore, clinical evidence is lacking for amifostine, superoxide dismutase, protein supplements, sucralfate, and antioxidants (including vitamin E) [15].

Anti-diarrheal agents are generally used in the treatment of ARE. Severe diarrhea often persists in many patients despite these medications. Moreover, these agents cannot be used as prophylaxis to reduce ARE because they often cause constipation. The somatostatin analogue, octreotide is reported to be effective in the treatment of chemotherapy-induced diarrhea [16]. Chemotherapy agents include 5-FU, cisplatin, methotrexate, doxiorubicin and irinotecan. Regarding ARE, Yavuz *et al.* [17] conducted a randomized controlled trial of octreotide for ARE. In the trial, octreotide was compared with diphenoxylate hydrochloride plus atropine sulphate. Diarrhea resolved more quickly and a decrease was observed in the number of patients needing to discontinue pelvic radiotherapy in the octreotide arm. ARE was completely resolved in 61% of the octreotide group and 14% in the control group. Thus, these agents have potential in the treatment of ARE if cost is not considered. The burden of treatment costs cannot be ignored, particularly in patients with malignant tumors, because treatment costs for malignancy are generally high. ARE typically does not persist for a long time after radiotherapy. Considering these things, the cost of octreotide for ARE is slightly expensive ($70 or 7800 yen/day). In addition, octreotide is administered via a subcutaneous injection, which may be burdensome for patients.

The mechanisms responsible for the development of ARE have been suggested to include the cyclooxygenase pathway mediating tissue injury and pain through the upregulation of pain-evoking prostaglandin E2 (PGE2) and proinflammatory cytokines [5]. The radicals generated by radiation destroy intestinal crypt epithelial cells, which leads to mucosal injury. When a large proportion of crypts in the intestines are destroyed, normal barrier function is lost, thereby exposing the normally sterile lamina propria to luminal microbes. This triggers an acute inflammatory response associated with immune cellular infiltrates (T-lymphocytes, macrophages and neutrophils), causing the loss of epithelial cells as well as degradation of the extracellular matrix in the lamina propria due to the enzymes and mediators released by immune cells. TJ-14 suppresses the production of PGE2, interleukin (IL)-6, and IL-8 in lipopolysaccharide-treated human gingival fibroblasts [7]. The gene expression of cyclooxygenase 2, cytosolic phospholipase A2, and PGE synthase was also shown to be decreased following an exposure to TJ-14. Additionally, TJ-14 exhibits multiple antioxidative abilities [8]. The main antioxidative effect of TJ-14 appears to be due to the combination of reactive oxygen species scavenging, its role as a reductant, and some minor oxidative activities. Therefore, TJ-14 appears to protect the gastrointestinal mucosa from radiation-induced inflammation by downregulating pro-inflammatory prostaglandins in the cyclooxygenase pathway.

In this clinical study, the TJ-14 efficacy for ARE was observed in 86% of patients. According to previous clinical trials, similar to octreotide, TJ-14 effectively prevented diarrhea associated with chemotherapy. Mori *et al.* [6] performed a randomized trial on advanced small cell lung cancer to elucidate the effects of TJ-14 on the reduction of Grade 3 or higher diarrhea caused by treatments with cisplatin/irinotecan. Chemotherapy-induced diarrhea occurred in 1 out of 18 patients in the TJ-14 group, but in 10 out of 23 in the control group (*P* = 0.02). Similar findings were reported for diarrhea as an adverse effect of epidermal growth factor receptor–tyrosine kinase inhibitor (afatinib) for non-small cell lung cancer [18]. TJ-14 is generally taken orally and directly acts on intestinal mucosal cells. Based on its biological and clinical background, TJ-14 exerts positive therapeutic effects on ARE. The efficacy of TJ-14 against ARE in the present clinical study was similar to that in the previous octreotide trial. TJ-14 non-hematological toxicities were Grade 1 or less in the present study. Moreover, TJ-14 only costs 60 US cents or 66 yen/day, which is less than one thousandth the cost of octreotide. Therefore, TJ-14 has potential as a standard treatment for ARE. A randomized double-blind placebo-controlled study to reconfirm the effects of TJ-14 is currently being planned.

## Supplementary Material

MuraiJRR0314_supupplement_material_rrz025
