# Supplementary material for: Efficacy of herbal medicine TJ-14 for acute radiation-induced enteritis: a multi-institutional prospective Phase II trial
Source: J Radiat Res. 2019 Nov 6;61(1):140–5. doi: 10.1093/jrr/rrz025 (PMC7022136; doi:10.1093/jrr/rrz025)
Supplement: MuraiJRR0314_supupplement_material_rrz025 [file muraijrr0314_supupplement_material_rrz025.docx]

**Supplemental material 1. TJ-14 ingredients**

TJ-14 is composed of 7 crude herbs in fixed proportions: 5 g of Pinellia Tuber, 2.5g of Scutellaria Root, 2.5g of Processed Ginger, 2.5 g of Glycyrrhiza, 2.5 g of Jujube, 2.5 g of Ginseng and 1 g of Coptis Rhizome.

**Supplemental material 2. Research facilities and patient numbers**

|  | ***Patient number*** |
| --- | --- |
| ***Nagoya City University Hospital*** | 12 |
| ***Gifu University Hospital*** | 7 |
| ***JCHO Chukyo Hospital*** | 1 |
| ***Okazaki City Hospital*** | 2 |
|  |  |

**Supplemental material 3. Patient self-reporting system**

**Body weight**  kg

**1. How frequently did you defecate yesterday?**  times/day

**2. What was the consistency of the stool?**

1) watery stool 2) muddy stool, like rice milk

3) hard stool, like mashed potato 4) hard and dry stool

**3. How frequently did you take the herbal medicine yesterday?** times/day

**4. If you have abdominal pain, indicate its intensity on the figure below.**

**The 11-point numeric scale ranges from '0' representing one pain extreme (*NO PAIN*) to '10' representing the other pain extreme (*WORST POSSIBLE PAIN*).**


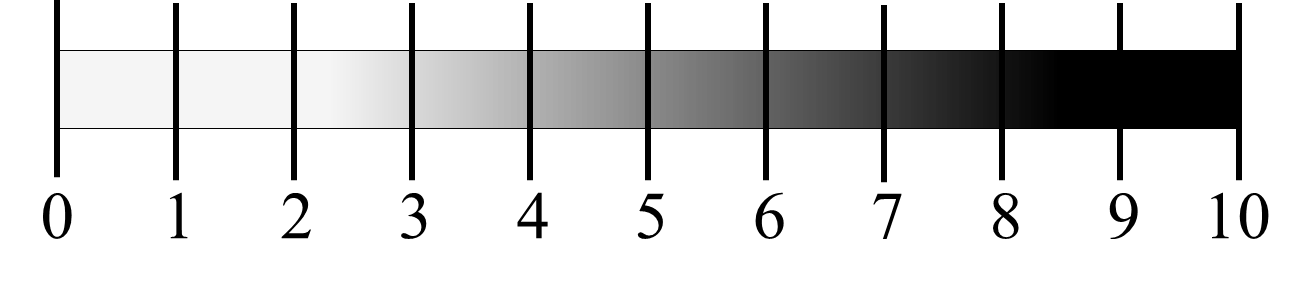


**5. How long did the abdominal pain last?**

(1-6 hours, 6-12 hours, 12-24 hours, No symptoms)

**6. Did the pain or diarrhea disturb your sleep?**  ( Yes / No )

**7. Does abdominal gas disturb you?** ( Yes / No )

**8. Does abdominal distension disturb you?**  ( Yes / No )

**9. Do you feel general weariness all day?**  ( Yes / No )

**10. Do you have an appetite?**  ( Yes / No )
